# LSTM-based prediction method for shape error of steel truss during incremental launching construction

**DOI:** 10.1371/journal.pone.0324932

**Published:** 2025-07-10

**Authors:** Zhe Hu, Hao Chen, Chunguang Dong, Qinhe Li, Ronghui Wang

**Affiliations:** 1 School of Civil Engineering and Transportation, South China University of Technology, Guangzhou City, Guangdong Province, China; 2 First Branch Company, Poly Changda Engineering Co., Ltd., Guangzhou City, Guangdong Province, China; University of Zanjan, IRAN, ISLAMIC REPUBLIC OF

## Abstract

Accurate shape control during steel truss incremental launching remains a persistent challenge in bridge engineering, primarily due to dynamic geometric variations induced by continuous spatial translation. Conventional measurement-based approaches often lead to inaccurate error determination and insufficient control criteria due to continuous geometric variations during structural movement. This study presents a novel Long Short-Term Memory (LSTM)-based methodology for real-time prediction and adaptive control of shape errors in launching processes. First, an error matrix is established based on the actual pushing measurement plan, and numerous error splines are generated using virtual assembly technology. These splines are output as an error matrix and encoded into a machine-readable format, leading to the establishment of a sliding window method for recursive prediction and updates of the predictions (measured values). Then, the LSTM model is trained using this sliding window approach, achieving a root mean square error(RMSE) of 0.03 on the test set. Field experiments demonstrate that the predicted values from the LSTM model closely align with the measured values, maintaining short-term shape error prediction accuracy within 3 mm. However, prediction accuracy diminishes for longer time steps as the step length increases. Following model updates with measured data, the accumulated prediction error rapidly decreases. The proposed prediction method for shape errors during pushing exhibits high accuracy and versatility in similar projects, significantly reducing time spent on manual error handling and minimizing computational inaccuracies.

## 1. Introduction

In 1959, when incremental launching construction technology was first applied to the Ager Bridge in Austria, it proved to be both economical and efficient. After over 30 years of continuous development, incremental launching remains the predominant bridge construction method worldwide. Some notable projects that utilize this technique include the Millau Viaduct in France (2004) [1], Pavilion Bridge in Spain (2011) [2], Ilsun Bridge in South Korea (2011) [3], Leigh Road Bridge in the UK (2016) [4], and the Jinan Huanghe rail-cum-road bridge in China (2020) [5]. While incremental launching offers advantages such as lightweight construction, flexibility, and the ability to cross terrain barriers [6,7], it also introduces unique challenges in monitoring cumulative assembly errors, particularly due to dynamic structural deformations during the incremental launching phase [8].Conventional discrete measurements, typically conducted at predetermined stages before final positioning, prove inadequate for tracking time-dependent deformations arising from multiple interacting factors [9]. This limitation leads to significant discrepancies between interim monitoring data and actual structural geometry at completion, compromising construction quality control.

**Fig 1:**
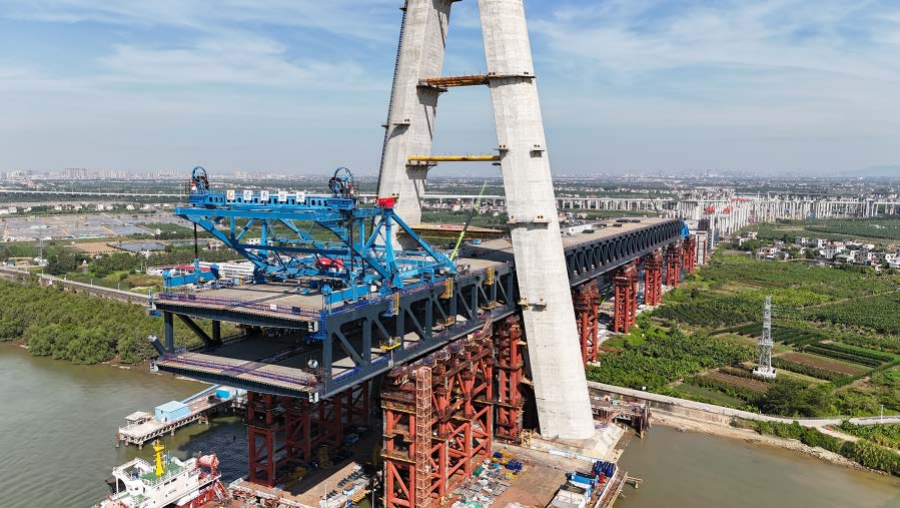
Process of steel truss incremental launching.

As revealed in Fig 1, the continuous deformation of steel trusses during incremental launching creates inherent difficulties in quantifying cumulative assembly errors. The practice of acquiring direct measurements exclusively during specific pre-positioning stages generates discrete error datasets that fail to represent ongoing structural changes, ultimately resulting in substantial deviations between interim measurements and final geometric errors. Four primary mechanistic factors drive this phenomenon:

(1) **Thermal deformation:** The steel truss-system experiences differential thermal expansion due to daily temperature variations [10]. The structural steel’s coefficient of thermal expansion induces anisotropic thermal strains, particularly pronounced in continuous truss configurations, generating unignorable dimensional discrepancies. (Nighttime measurements are proposed to mitigate thermal gradient effects.)
(2) **Dynamic support reaction evolution:** During the incremental launching process, the reaction forces and positions of the temporary supports for the steel truss continuously change, resulting in inaccuracies in the theoretical calculated deflection that should be deducted from the measured shape.
(3) **Fabrication tolerances:** The actual manufacturing precision of the steel truss is often lower than officially advertised, which adversely affects the measurement results of assembly errors at the bridge site [11].
(4) **Constructional constraints**: The removal of temporary connection shims at the splice joints and residual welding stresses can also impact the final positioning of the beam sections.

Finite element modeling and geometric control methods have been developed for the shape error control of bridges during incremental launching construction. C. Li et al. (2014) [12] proposed a phase-transformation method that achieves coordinate conversion through rigid-body displacement of the stress-free alignment of steel girders, thereby enabling adaptive alignment control during incremental launching processes. Z. Hu et al. (2015) investigated the stress and deformation levels of continuous hybrid arch-girder bridges during incremental launching, revealing that the temporary steel arch-girder connectors and wind loads critically influence structural safety. S. Ding et al. (2021) [5] analyzed the jacking process of continuous steel truss girders using a combination of the incremental launching method and a synchronous control system, proposing the strategy of adopting elastic bracing pads to reduce the unfavorable effects of height differences in the main trusses. H. Martins et al. (2025) [13] demonstrated that incrementally launched steel trusses with modular bolted moment joints enhance erection efficiency through kit-of-parts assembly and structural redundancy, validated by staged shell-element FE modeling compared to conventional frame analysis to guide design. These studies provide valuable theoretical and engineering insights into collocation errors, but they do not address the problem of recovering missing measurement data.

In recent years, Deep learning has emerged as a transformative methodology in civil engineering, demonstrating particular efficacy in construction and structural health monitoring (SHM) [14] through its capacity to decode complex spatiotemporal patterns from multi-modal sensor data streams. J. Zhang et al. (2024) [15] systematically introduced the mainstream methods for indeed signal recovery in the field of SHM, including finite element methods, sparse representation methods, statistical inference methods, machine learning algorithms, and then demonstrated the recovery process of missing measurement data through the case of Hardanger Bridge in Norway; Huang et al. (2023) [16] developed a two-stage method combining element relative MSE and sailfish optimization for bridge bearing damage identification, achieving 95.2% accuracy in multiscale validations (numerical to I-40 Bridge), bridging a critical gap in structural health monitoring; also, M. Huang et al. (2024) [17] developed an SE-enhanced CNN-BiGRU hybrid network to resolve spatiotemporal feature decoupling in SHM data reconstruction, demonstrating 33% R² improvement and 93% NRMSE reduction under environmental interference through validation on the Guangzhou Tower, outperforming conventional CNNs. The above examples certainly illustrate the successful application of deep learning in civil engineering.

In general engineering measurement, while repeated testing methods effectively eliminate random errors, manual identification and correction of systematic errors remain operationally challenging due to their inherent correlation with measurement system configuration. When isolating errors proves difficult, a large volume of data from repeated observations can assist neural networks in capturing the necessary information for error elimination. Long Short-Term Memory (LSTM) [18]networks can overcome the independence of each measurement sample and establish temporal relationships between sample sequences. This capability allows the LSTM model to consider how errors in previously assembled beam sections impact subsequent sections, while also accounting for the influence of errors in later sections on earlier ones. Consequently, this bidirectional transmission of error accumulation enhances the accuracy of shape error predictions during the pushing process of the steel truss.

While existing literature demonstrates growing applications of LSTM networks in structural engineering, their implementation for real-time control during construction phases remains notably underexplored. Especially in incremental bridge launching processes, the research to find an LSTM-based control method is even more challenging. In SHM systems, well-defined indicators and continuous records are comprehensively stored as spatiotemporal datasets through embedded sensors. This data structure renders Long Short-Term Memory (LSTM) networks particularly effective for SHM applications due to their proven capability in modeling sequential dependencies. Miao et al. (2021) [19] leveraged LSTM to model bridge degradation through 12 critical factors derived from 3,368 bridges, achieving superior accuracy over traditional multilayer perceptron. This approach establishes a paradigm for multi-parameter-driven deformation prediction, which is particularly relevant to truss shape error analysis during phased construction. For real-time monitoring scenarios; Wang et al. (2022) [20] developed an LSTM framework that correlates temperature variations with main girder deflection, demonstrating how time-dependent environmental factors can be systematically integrated into deformation prediction models; Meng et al. (2023) [21] further enhanced prediction precision through hybrid signal processing, combining Empirical Mode Decomposition with LSTM to effectively denoise structural deformation data from large-span bridges. Notably, these applications consistently emphasize LSTM’s advantage in processing non-stationary sequential data, which is a critical feature for predicting time-varying shape errors during dynamic construction processes. Recent advances in LSTM optimization demonstrate its efficacy for dynamic structural analysis, Greff et al. (2017) [18] established LSTM architecture optimization principles through variant benchmarking – both critical for handling time-varying construction data like truss pushing errors; Xiao et al. (2022) [22] demonstrated LSTM’s capacity to model environmental-mechanical interactions through steel member thermal response prediction. These developments confirm LSTM’s superiority in processing non-stationary sequential patterns inherent to dynamic construction processes.

Based on this literature review, it is evident that LSTM applications in the field of bridge construction control are scarce. The method proposed in this paper for predicting and controlling assembly errors during the pushing process of steel trusses, based on LSTM, represents an innovative application with practical and economic value for improving construction control efficiency.

## 2. Establishment of the error spline library

### 2.1 Establishment of the error matrix

During the construction process of the steel truss, significant changes occur in its geometry as the guiding beam is initially positioned onto the pier and progressively pushed to its final configuration. Despite the continuous change in the steel truss shape, the system remains inherently stress-free. This indicates that the geometric deviations arising from the initial assembly matching process remain invariant throughout the operation. Specifically, the incremental launching construction methodology involves the transportation of the fully assembled steel truss to its predetermined design location. As such, the geometric inaccuracies or deviations are solely attributed to the alignment discrepancies between the newly installed segment and the existing structure during the lifting process on the assembly platform. Notably, the transportation process itself does not induce any additional geometric variations, as the truss structure preserves its pre-assembled configuration.

**Fig 2:**
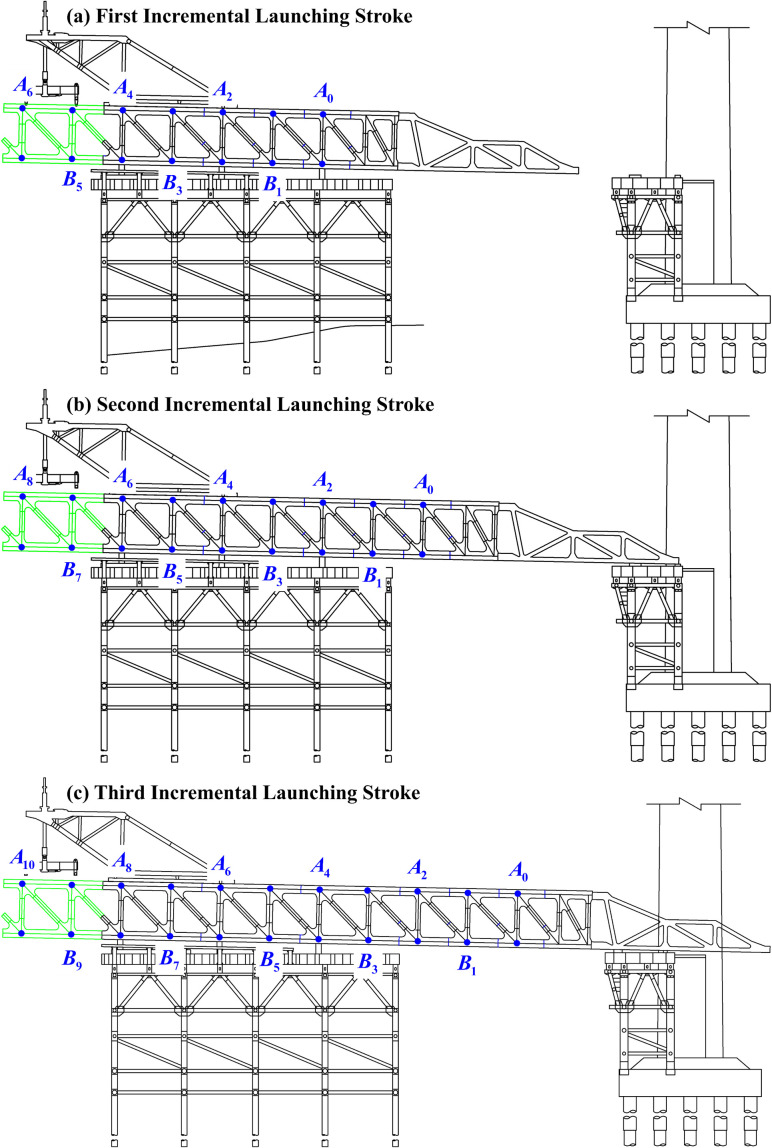
Process of steel truss guiding beam onto support piers.


In addition to constructing an assembly platform with sufficient working space, the pushing process requires temporary pier supports. The assembly platform is used to lift and assemble beam segments according to the manufacturing shape specified by the factory, while the temporary pier supports provide vertical support for the continuous transport of large beam segments during the pushing process. Fig 2 illustrates the process of pushing the guiding beam onto the pier for the steel truss. Typically, each beam segment corresponds to a complete span, with cantilever components available for assembly at both ends. Once a new segment is assembled, two complete spans are formed between the new and previous segments. Therefore, the length of the assembly platform must accommodate an even number of spans. Let the first upper chord node of the standard segment be denoted as *A*0. If the assembly platform can accommodate *j* + 2 nodes for shape verification, the elevation measurement data for the first, second, and third rounds of shape verification are as follows:

$$AH_{1}=\left[\;\;\;\begin{array}{cccc} {}*20cAH_{1}^{0} & AH_{1}^{1} & ... & AH_{1}^{j+1} \end{array}\;\;\;\right]$$ (1)

$$AH_{2}=\left[\;\;\;\begin{array}{cccc} {}*20cAH_{1}^{2} & AH_{1}^{3} & ... & AH_{1}^{j+1} \end{array}\;\;\;\right]$$ (2)

$$AH_{3}=\left[\;\;\;\begin{array}{cccc} {}*20cAH_{1}^{4} & AH_{1}^{5} & ... & AH_{1}^{j+5} \end{array}\;\;\;\right]$$ (3)

When *j* = 5, the nodes for the first three rounds of shape verification are [*A*_0_ ~ *A*_6_], [*A*_2_ ~ *A*_8_], and [*A*_4_ ~ *A*10]. If the entire incremental launching process involves *k* rounds of shape verification, the elevation measurement data can be organized in matrix form as follows:


$$AH=\left\{\;\;\;\;\begin{array}{c} {}*20cAH_{1}\\ AH_{2}\\ \vdots \\ AH_{k} \end{array}\;\;\;\right\}=\left[\;\;\begin{array}{cccc} {}*20cAH_{1}^{0} & AH_{1}^{1} & \cdots & AH_{1}^{j+1}\\ AH_{2}^{2} & AH_{2}^{3} & \cdots & AH_{2}^{j+3}\\ \vdots & \vdots & \ddots & \vdots \\ AH_{k}^{0+2(k-1)} & AH_{k}^{1+2(k-1)} & \ldots & AH_{k}^{j+1+2(k-1)} \end{array}\;\;\;\right]$$ (4)

Before conducting shape verification, it is necessary to standardize both the measurement data and the factory-set manufacturing shape data, as their overall placement angles may differ. Taking the elevation data from the first round of verification as an example:

Initial positioning radius:


$$R_{1}^{j}=\sqrt{{\left(AX^{j}-AX^{0}\right)}^{2}+{\left(AH_{1}^{j}-AH_{1}^{0}\right)}^{2}}$$ (5)

Initial positioning angle:


$$\alpha_{1}^{j}=\arctan\left(\frac{AH_{1}^{j}-AH_{1}^{0}}{AX^{j}-AX^{0}}\right)$$ (6)

The rotation angle to make nodes *A*_0_ and *A*_(j-1)_ at the same elevation:


$$\theta_{1}=\arctan\left(\frac{AH_{1}^{j-1}-AH_{1}^{0}}{AX^{j-1}-AX^{0}}\right)$$ (7)

The elevation of the j-th node after standardization:


$$SAH_{1}^{j}=R_{1}^{j}\sin(\alpha_{1}^{j}-\theta_{1})-AH_{1}^{0}$$ (8)

Let $AX^{j}$ be the x-axis coordinate in the mileage direction of the upper chord at the j-th node. Since the difference between each measured value and the design value is small, a fixed design value can be utilized. The elevation matrix, along with and the multi-round verification data, can then be reorganized as follows:


$$AH^{S}=\left\{\begin{array}{c} {}*20cAH_{1}^{S}\\ AH_{2}^{S}\\ \vdots \\ AH_{k}^{S} \end{array}\right\}=\left[\begin{array}{ccccccc} {}*20c0 & AH_{1}^{S,1} & \cdots & AH_{1}^{S,j-2} & 0 & AH_{1}^{S,j} & AH_{1}^{S,j+1}\\ 0 & AH_{2}^{S,3} & \cdots & AH_{2}^{S,j} & 0 & AH_{2}^{S,j+2} & AH_{2}^{S,j+3}\\ \vdots & \vdots & \ddots & \vdots & \vdots & \vdots & \vdots \\ 0 & AH_{k}^{S,1+2(k-1)} & \cdots & AH_{k}^{S,j-2+2(k-1)} & 0 & AH_{k}^{S,j+2(k-1)} & AH_{k}^{S,(j+1)+2(k-1)} \end{array}\right]$$ (9)

Using the same method, the standardized manufacturing shape data matrix $H^{S}$ can be obtained. By subtracting the corresponding elements of the two elevation data matrices, the error matrix for the upper chord nodes can be calculated as follows:


$$AE=AH^{S}-H^{S}=\left[\begin{array}{c} {}*20cAE_{1}\\ AE_{2}\\ \vdots \\ AE_{k} \end{array}\right]$$ (10)

$$\left[\begin{array}{c} {}*20cAE_{1}\\ AE_{2}\\ \vdots \\ AE_{k} \end{array}\right]=\left[\begin{array}{ccccccc} {}*20c0 & AE_{1}^{1} & \cdots & AE_{1}^{j-2} & 0 & AE_{1}^{j} & AE_{1}^{j+1}\\ 0 & AE_{2}^{3} & \cdots & AE_{2}^{j} & 0 & AE_{2}^{j+2} & AE_{2}^{j+3}\\ \vdots & \vdots & \ddots & \vdots & \vdots & \vdots & \vdots \\ 0 & AE_{k}^{1+2(k-1)} & \cdots & AE_{k}^{j-2+2(k-1)} & 0 & AE_{k}^{j+2(k-1)} & AE_{k}^{\left(j+1)+2(k-1\right)} \end{array}\right]$$ (11)

### 2.2 Twin spline library

In practical engineering, the shape error data obtained through measurement is often limited, making it insufficient for training an LSTM model. However, by simulating shape error generation on a computer, a vast dataset can be created. Using transfer learning, the LSTM model can then be adapted to process real engineering data effectively. The digital twin technology approach for shape error involves several steps. First, generate the geometric model of the beam segments based on their length and height from the drawings. Next, set the assembly error angle (*α*) between the beam segments, enabling the calculation of node coordinates under various combinations of positive and negative error angles. Finally, record the spline coordinate information in the error matrix. By incorporating relevant content from Section 3, a machine-readable format can be generated, preparing the dataset for LSTM model training.

**Fig 3:**
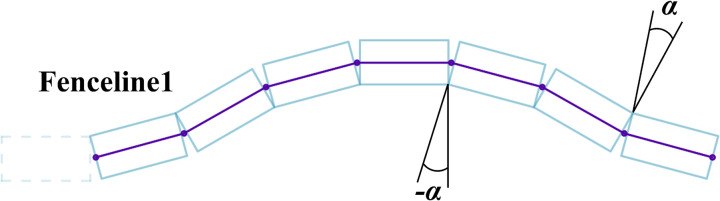
Assembly error angle of a single spline.


Fig 3 shows the assembly error angle α of a single spline, which is generally set according to the following formula:

α=atan(Δ/L) (12)

In the formula,Δ represents the maximum vertical assembly error between beam segments, which can be determined through test assembly measurements;L is the length of the beam.

**Fig 4:**
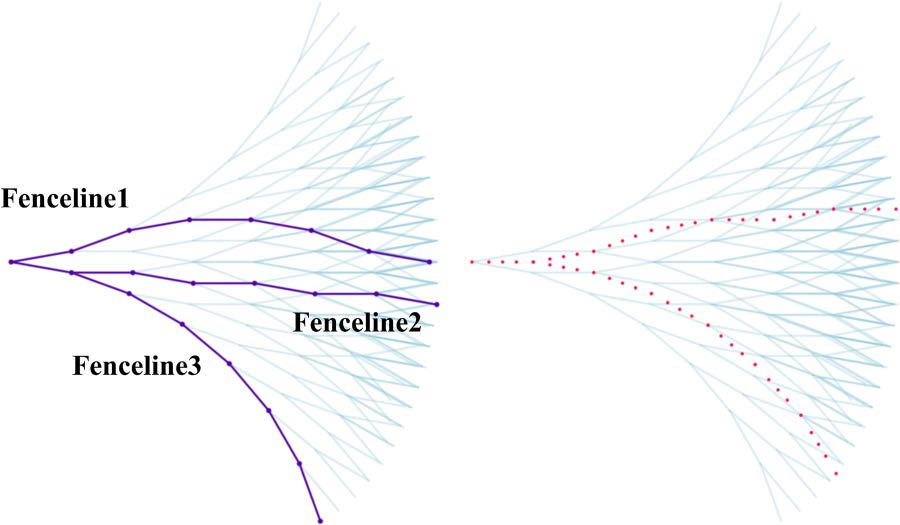
Generated spline library.

If the total number of simulated beam segments is *Bn*, and each segment has two types of assembly error angles, the total number of generated error splines will be 2^*Bn*^. As shown in Fig 4, the mesh funnel consists of seven assembly beam segments, resulting in a total of 2^7^ = 128 splines.

In the error spline library, Fenceline1 represents an approximate arch with equal elevation at both ends, while Fenceline2 is a polyline with alternating positive and negative error angles, generally following a straight trajectory. Fenceline3, on the other hand, is a curved line with a continuously declining elevation. The red dashed line, which crosses the blank areas of the grid, illustrates a possible on-site error distribution scenario. Although this line is not directly captured by the LSTM model, the learning patterns of nearby splines provide valuable computational references. This suggests that, in terms of data semantics, using fixed data formats with non-differentiated numbers can enable a mutually adaptable computational approach.

### 2.3 Automatic error verification window

Equation (13) is a simplified form of equation(11). The error matrix AE has a well-defined physical meaning: each row represents the total number of verification rounds, while each column corresponds to the number of nodes in each verification. However, the data within this matrix is not easily interpretable by machine learning algorithms. Therefore, the next step is to develop a more machine-friendly representation, enabling automated error verification within the sliding window framework.

As previously established, the total number of verification rounds is *k*, and each verification involves *j* + 2 nodes. Thus, the total number of unique nodes, excluding repeated verifications, is p = j + 2k. Additionally, each time a beam segment is pushed, two nodes exit the verification area, meaning any single node can be verified up to *q* times, where *q* = [(*j* + 2)/2], with ceil indicating rounding up to the nearest integer. To facilitate machine recognition, a blank table $AE^{fea}(p\times q)$ is created (Filled with 0). The notation ae(m,n) represents an element at row m and column n in $AE^{fea}(p\times q)$, corresponding to an element AE(e,f) in the error matrix AE.


$$AE=\left[\begin{array}{ccc} {}*20cAE(1,1) & \cdots & AE(1,j+2)\\ \vdots & AE(e,f) & \vdots \\ AE(k,1) & \cdots & AE(k,j+2) \end{array}\right]$$ (13)

$$AE^{fea}=\left[\begin{array}{ccc} {}*20cae(1,1) & \cdots & ae(1,q)\\ \vdots & ae(m,n) & \vdots \\ ae(p,1) & \cdots & ae(p,q) \end{array}\right]=\left[\begin{array}{ccc} {}*20c0 & \cdots & 0\\ \vdots & \ddots & \vdots \\ 0 & \cdots & 0 \end{array}\right]$$ (14)

**Fig 5:**
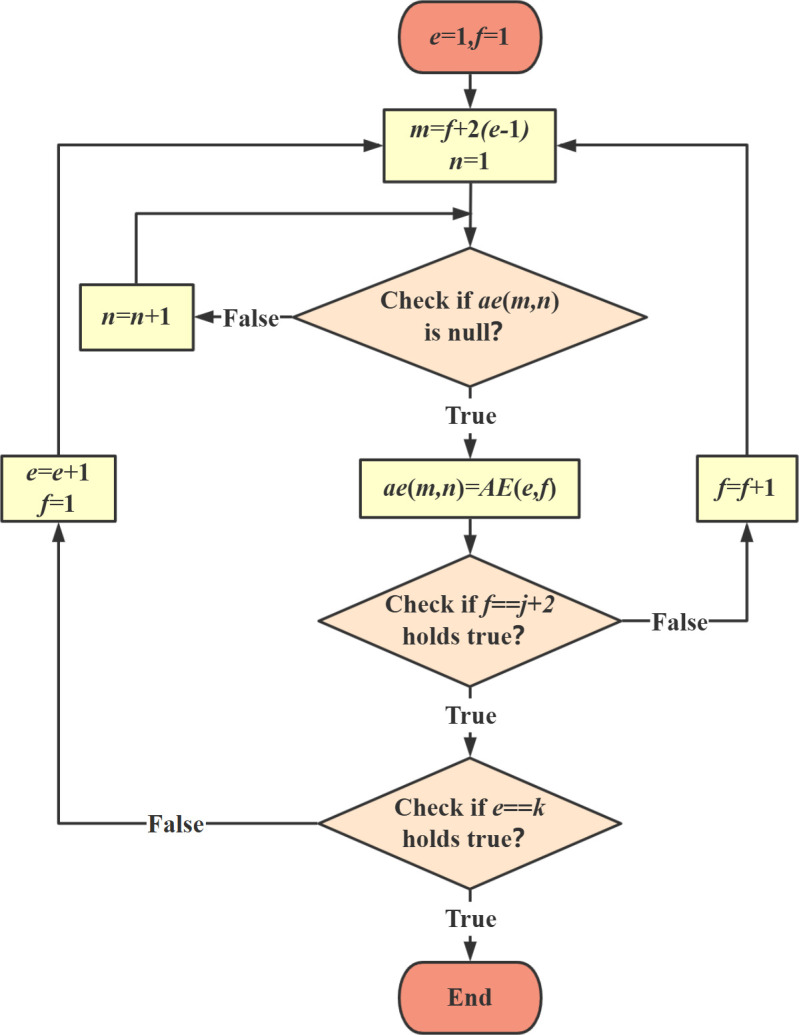
Assembly process of machine recognition form.


In Fig 5, the elements of the error matrix AE are arranged into the machine-recognizable form $AE^{fea}$ according to the flowchart requirements. This arrangement results in a feature matrix with a sequence length of p and a feature quantity of *q*. Additionally, the previously introduced error prediction array $AE^{lab}$ is incorporated alongside the feature matrix $AE^{fea}$ in the machine recognition form $AE^{learn}$.

$$AE^{learn}=AE^{fea}AE^{lab}$$ (15)

**Fig 6:**
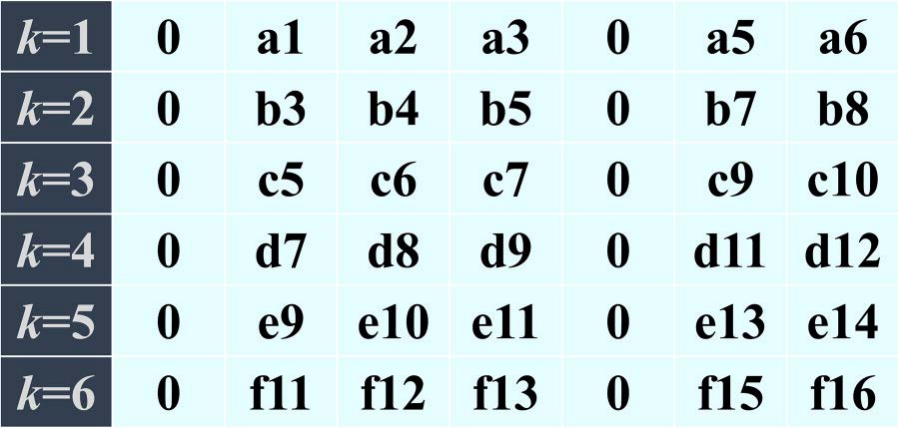
Original record form of error matrix.

**Fig 7:**
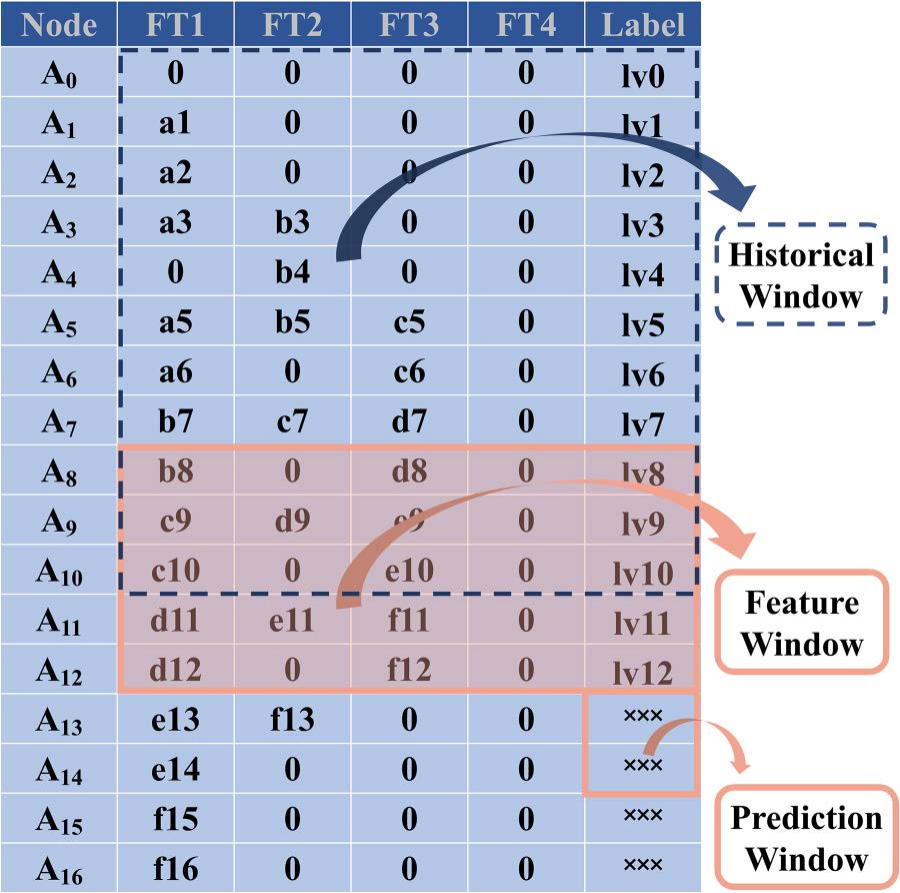
Form for machine recognition.

In a construction process requiring *k* = 6 rounds of shape verification with 7 nodes per round, the initial dataset is presented in Fig 6. Following the processing methodology outlined in Fig 5, the data is transformed into a structured format suitable for machine learning and recognition, as illustrated in Fig 7. The resulting feature matrix exhibits a sequence length of *p* = 5 + 2 × 6 = 17 nodes, derived from the sum of the constant term and the product of the number of iterations and features. The number of features is determined as *q* = ceil [(5 + 2)/2] = 4, which signifies that data from 4 rounds of verification per node is utilized. To ensure consistency within the matrix, any missing data points are imputed with zeros. This approach maintains the integrity and uniformity of the dataset, facilitating accurate feature extraction and analysis.

In practical feature engineering applications, feature 4(FT4) demonstrates limited informational value due to its high zero-value prevalence, potentially offering negligible contribution to model performance enhancement. To optimize the feature space, three viable modifications warrant consideration: 1) augmentation through combination with nodal mileage coordinates {AX}; 2) strategic introduction of supplementary mechanical parameters such as member stress distribution or support reaction forces; 3) Manufacturing errors are also effective predictive evidence. Alternatively, feature elimination could be implemented to streamline computational efficiency without compromising model efficacy.

**Fig 8:**
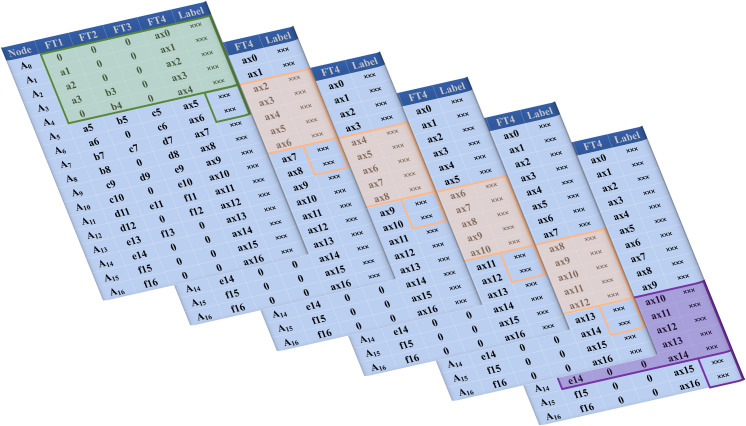
Sliding window process.

Fig 8 depicts the operational mechanism of the sliding window algorithm. This methodology operates by utilizing historical verification-phase data to forecast subsequent outcomes. The neural network processes temporal feature window data and generates predictive outputs within the specified prediction window. Prediction error is quantified through residual analysis between model outputs and corresponding ground truth labels. An iterative parameter optimization process is subsequently executed to progressively reduce the prediction discrepancy.

During batch processing of sliding window operations, preventing potential prediction value leakage becomes paramount. The feature window should strictly exclude direct inclusion of target answers, as such inclusion would inherently result in information contamination. While the feature window may incorporate current-round ground truth labels as contextual features, it must rigorously exclude any reference to subsequent-round label values to maintain temporal integrity.

## 3. Comprehensive error prediction

The arrangement of shape errors along the longitudinal direction of the bridge effectively forms a sequence of coordinate axes, making the LSTM’s long-term and short-term memory processing particularly suitable for error propagation calculations. The errors in the assembled beam segments resemble long-term memory information, with the resulting overall joint angles determining the direction of errors in the segments yet to be assembled. During the matching process at the assembly interface, new relative errors—representing short-term memory information—are introduced. By incorporating long-term and short-term memory information, comprehensive error prediction results for each sequence point can be obtained.

**Fig 9:**
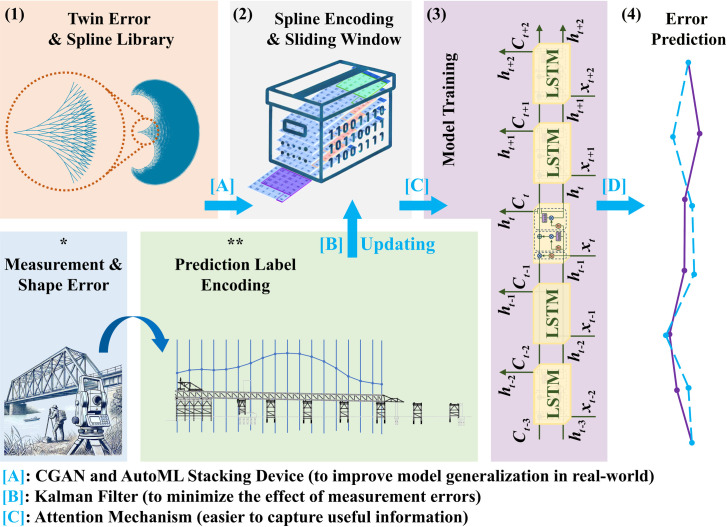
Comprehensive prediction process of shape errors.

The prediction of errors generally follows several steps, as illustrated in Fig 9:

(1) **Twin Error & Spline Library**: Twin error splines are generated using virtual assembly technology to create a comprehensive error database.
(2) **Spline Encoding & sliding window**: The errors in the spline database are encoded. To enable the machine to recognize and process the data, it is essential to appropriately determine the sliding window mode, including the window size and prediction step length.
(3) **Model Training**: The model’s training process primarily involves optimizing hyperparameters, including both architecture and training parameters, to maximize performance.
(4) **Error Prediction**: The trained model can predict shape errors during the construction process and provide guidance for shape control direction.

Finally, [A][B][C] is the module that improves model performance, as described in the following sections.

## 4. Model optimization

### 4.1 Architecture optimization

Generative Adversarial Networks (GAN), while predominantly employed for static data generation (e.g., images, text), require substantial architectural modifications for temporal forecasting applications. This section introduces a conditional GAN (CGAN)-based framework for sequential prediction. As an extension of conventional GAN architecture, CGAN integrates contextual conditions into both generator and discriminator inputs through conditional vectors, enabling the generation of temporally coherent sequences conditioned on historical patterns. For time-series forecasting tasks, the proposed implementation leverages past observational windows as contextual constraints to probabilistically estimate future temporal states.

**Fig 10:**
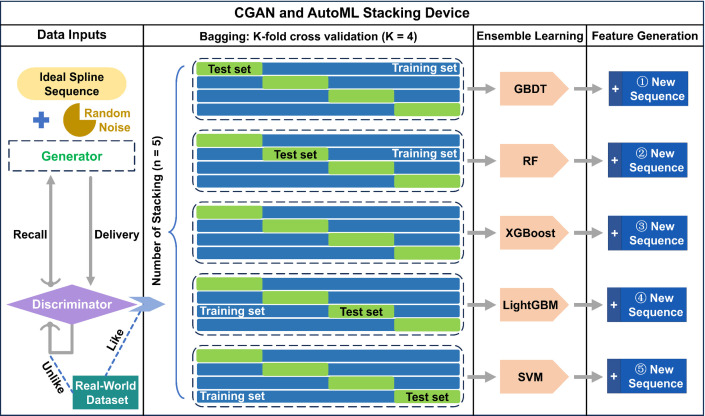
CGAN and AutoML stacking device.

To enable the LSTM model to capture more comprehensive and effective information, an automated machine learning module is incorporated to generate novel feature sequences. As illustrated in Fig 10, at the input stage, random noise is introduced by the generator to address the distortion discrepancy between spline sequences and real-world data. When the discriminator classifies the processed data as authentic real-world datasets, the input data undergoes bagging to form distinct training and testing sets. Conversely, if the discriminator identifies the data as non-authentic, the sequence is redirected back to the generator for reprocessing. The discriminator, serving as the core component of the generative adversarial network (GAN), functions fundamentally as a binary classifier. This design permits the implementation of various simple algorithms, including decision trees, for the binary classification task. The successful migration of real-world data information into spline-simulated data is achieved when the generator produces sufficiently realistic data to deceive the discriminator, resulting in misclassification of synthetic data as genuine.

The bagged data undergoes K-fold cross-validation to ensure the ensemble learner mitigates the impact of uneven dataset partitioning. Subsequently, multiple mainstream ensemble algorithms are stacked to construct base learners, which process raw sequential data to generate new feature sequences. Following preliminary evaluation through an attention module, the feature importance rankings reveal that GBDT, RF, XGBoost, LightGBM, and SVM demonstrate superior significance in feature generation. Consequently, the stacked architecture retains these five ensemble algorithms while eliminating others. Finally, through comprehensive consideration of computational efficiency, dataset scale, and base learner complexity, the K-value is optimized and set to 4 for cross-validation configuration.

**Fig 11:**
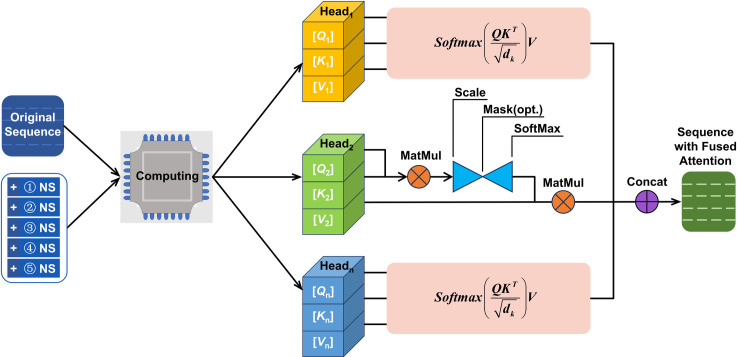
Attention mechanism.

The newly generated feature sequences and original sequences are subsequently integrated through an attention module for feature fusion, as depicted in Fig 11. The attention mechanism performs secondary encoding via generated multi-head [Q], [K] and [V] matrices, where the encoded representations explicitly quantify the relative importance of each feature value. This encoding enables the LSTM model to subsequently prioritize learning the most informative components. Through systematic evaluation of task complexity and computational constraints, the architecture ultimately implements an 8-head attention configuration for optimal performance.

### 4.2 Filter optimization

**Fig 12:**
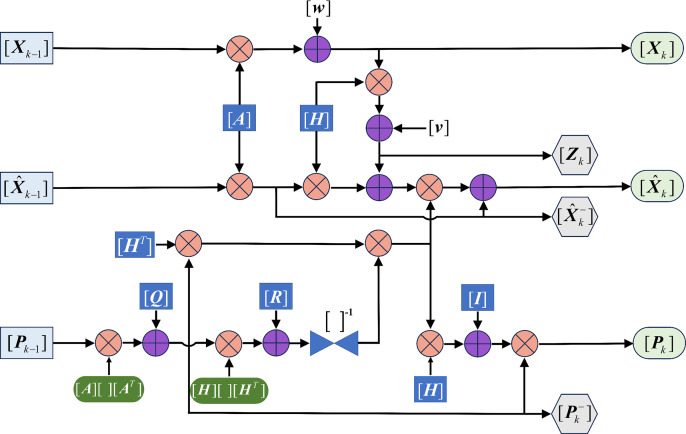
Kalman process.

As shown in Fig 12, the Kalman filtering process is used to quantify the uncertainty of the prediction. $\left[X_{k-1}\right]$, $\left[{\hat{X}}_{k-1}\right]$ and $\left[P_{k-1}\right]$ are input matrices that are filtered to output $\left[X_{k}\right]$, $\left[{\hat{X}}_{k}\right]$ and$\left[P_{k}\right]$. These output quantities are also recursive quantities, i.e., they will be fed into the system as input quantities again in the next round of filtering. $\left[Z_{k}\right]$, $\left[{\hat{X}}_{k}^{-}\right]$ and $\left[P_{k}^{-}\right]$ are intermediate process quantities that are not directly recursive themselves. The Kalman process undergoes complex matrix operations such as addition, multiplication, transposition, inversion, and higher conversion, and generally consists of two major steps: prediction and correction.

**Fig 13:**
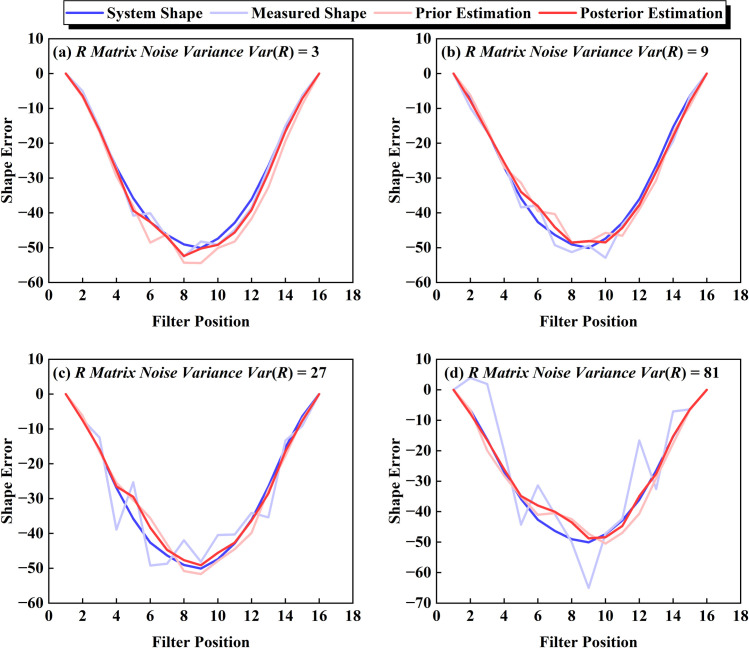
U-wave estimates versus R-matrix noise variance.

Within the Kalman Filter framework, system states and measurement values coexist with equivalent status, where their mutual calibration determines optimal estimation through weighted integration. This dual-source verification mechanism generally yields enhanced estimation accuracy compared to single-source approaches. In practical applications of large-segment assembly, manufacturing tolerances documented in factory reports serve as critical system references, while field-measured geometrical deviations at bridge construction sites constitute the measurement inputs. Notably, the system noise covariance (Q) in this context is significantly lower than the measurement noise covariance (R), as evidenced in Fig 13. The parametric analysis reveals that increased variance in the R matrix amplifies simulated measurement noise intensity. Nevertheless, the filtered estimates maintain coherent variation patterns with ground-truth system states, demonstrating the Kalman filter’s exceptional robustness against noise interference.

### 4.3 Hyperparameter optimization

Upon integration of optimized data from the sampler into the final architecture, hyperparameter optimization must be strategically initiated to maximize the fused model’s performance potential. Given the multidimensional nature of hyperparameters in deep learning architectures, exhaustive exploration of the combinatorial parameter space incurs prohibitive computational overhead. Therefore, comprehensive hyperparameter tuning is rarely implemented in practice. Should surplus computational resources become available for further optimization, this methodology necessitates preliminary subsampling of the full dataset for iterative training. Such stratified sampling reduces individual iteration durations (based on our GPU cluster benchmarks), while maintaining high correlation with full-dataset training outcomes, thus enabling efficient navigation through high-dimensional hyperparameter spaces.

**Fig 14:**
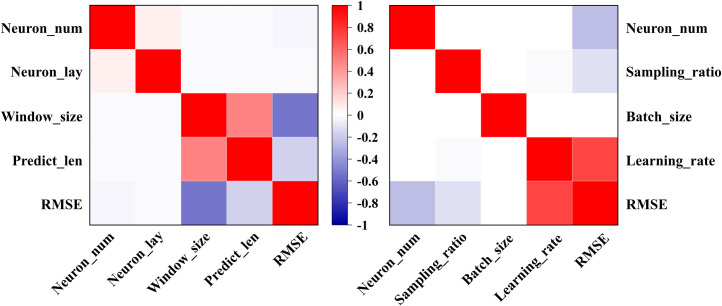
Correlation coefficient matrix.

In addition to sampling small datasets to reduce training costs, another approach is to reduce the dimensionality of the hyperparameter space. For example, if the grid size for the overall search is 4, having 8 superparameters would result in 4^8 model trainings, leading to significant time consumption. By dividing the hyperparameter space into two independent parts and training each part 4^4 times, search efficiency can be significantly improved. In this approach, neuron layers, window size, and prediction step length are grouped as architecture parameters, while sampling rate, batch size, and learning rate are categorized as training parameters. The number of hidden layer neurons is searched using a combination of random grid search. As shown in Fig 14, the learning rate, number of neurons, window size, and prediction step length are strongly correlated with the test set RMSE, making them key factors for optimizing model performance.

**Fig 15:**
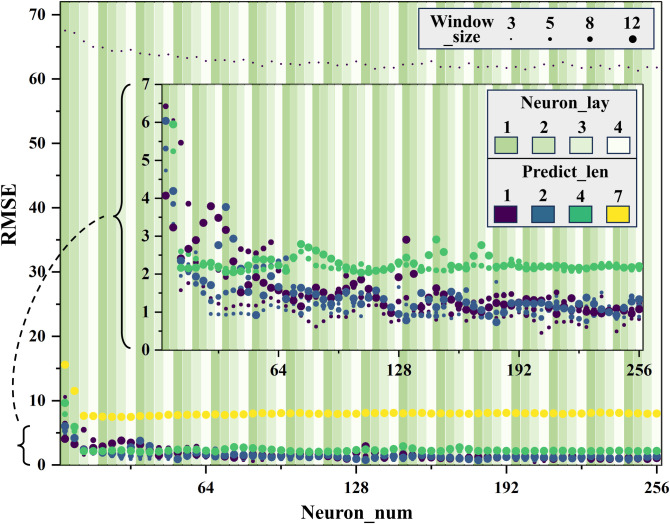
RMSE versus architectural parameters.

The distribution of the impact of architecture parameters on RMSE is shown in Fig 15. It can be observed that the number of neuron layers does not significantly improve prediction accuracy, so it is set to 1 to keep the architecture computationally efficient. For non-periodic sequence prediction tasks, such as predicting bridge shape errors, window sizes primarily capture temporal dependencies rather than periodic patterns. An empirical window size of 5 was selected to balance computational efficiency with sufficient information. Smaller windows (<5) risk omitting critical temporal dependencies (RMSE ≈ 63, Non-convergence), while larger windows (>5) introduce redundancy without improving prediction. Additionally, if the step length is too large, long-term prediction convergence is insufficient, as seen in the yellow and green scattered points in the figure. Notably, the scatter at the bottom (RMSE < 0.5) is composed of a window size of 5 and a step length of 1, which suggests the optimal sliding window mode.

**Fig 16:**
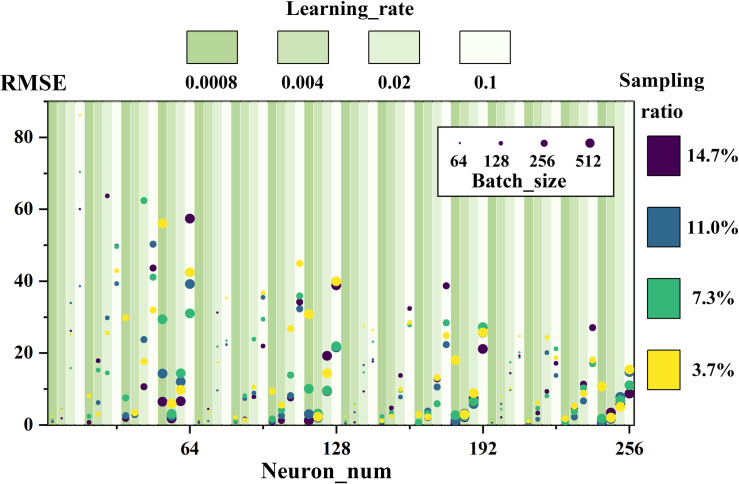
RMSE versus training parameters.

Fig 16 demonstrates the effect of the four Training parameters on the RMSE of the model, and it can be seen that the range of the RMSE is gradually narrowed with the increase of the number of neurons, which indicates that its effect on neural network learning is improved. In practical engineering projects, the number of neurons in the hidden layer should be set to the maximum allowed according to the size of the GPU memory capacity, so that the generalization ability of the model can be significantly improved. The batch size in the figure consists of scatter size, and there is no obvious correlation between them and RMSE, but in order to speed up the computation rate of the model, the batch size should be set to a larger value as much as possible. Sampling ratio are represented by scatter colors in the figure, and there is no differentiation between the effect on RMSE of a large proportion of sampling represented by dark dots and that of a small proportion of sampling in light colors, so as to indicate that the hyperparameters determined by the sampling model are equally adapted to the complete sample. Finally, when the learning rate is relatively small, i.e., the dark green raster in the figure, it is clear at this point that the scatter cluster of the RMSE is smaller overall. The learning rate, as the most sensitive parameter affecting the RMSE, should be used to do a precision grid search on top of the overall grid search.

**Fig 17:**
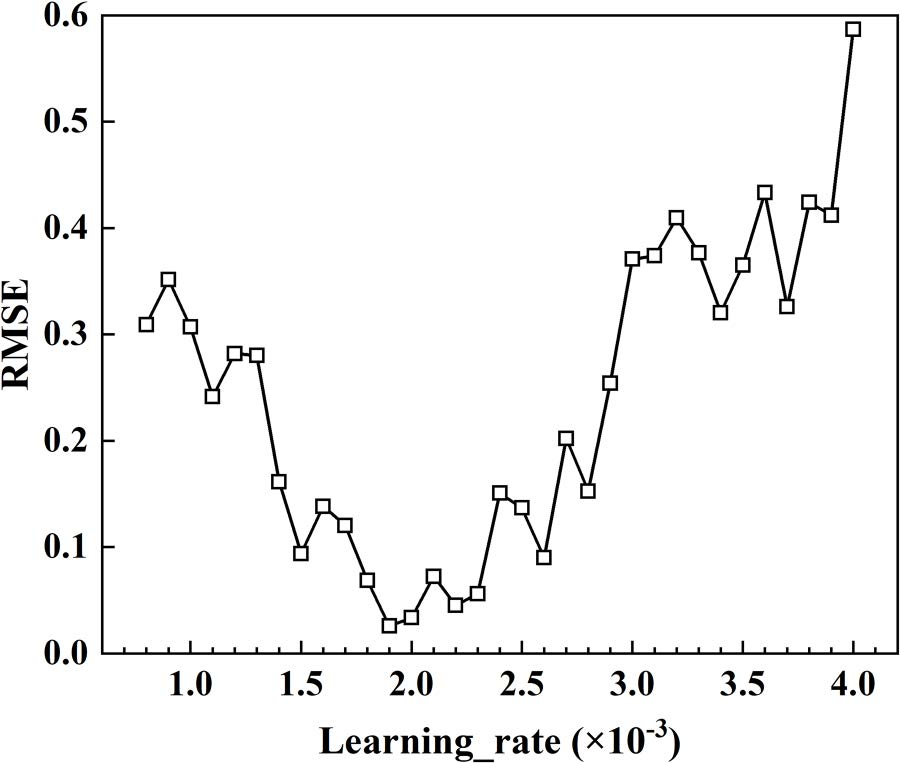
RMSE versus learning rate.

As shown in Fig 17, the optimized hyperparameters identified through overall grid search were systematically integrated into the final neural network architecture, with configuration parameters set as follows: Window_size = 5, Predict_len = 1, Neuron_num = 1000, Batch_size = 512. A high-resolution grid search was subsequently conducted for learning rate optimization within the interval [0.0008, 0.004], employing a precision threshold of 0.0001. Through rigorous evaluation, the model’s optimal learning rate was determined to be 0.0019. Final training conducted over 2,000 epochs with this optimized learning rate configuration achieved a minimum RMSE of 0.0315, representing the model’s peak performance metric.

## 5. Analysis of measured data and prediction results

### 5.1 Engineering background

**Fig 18:**
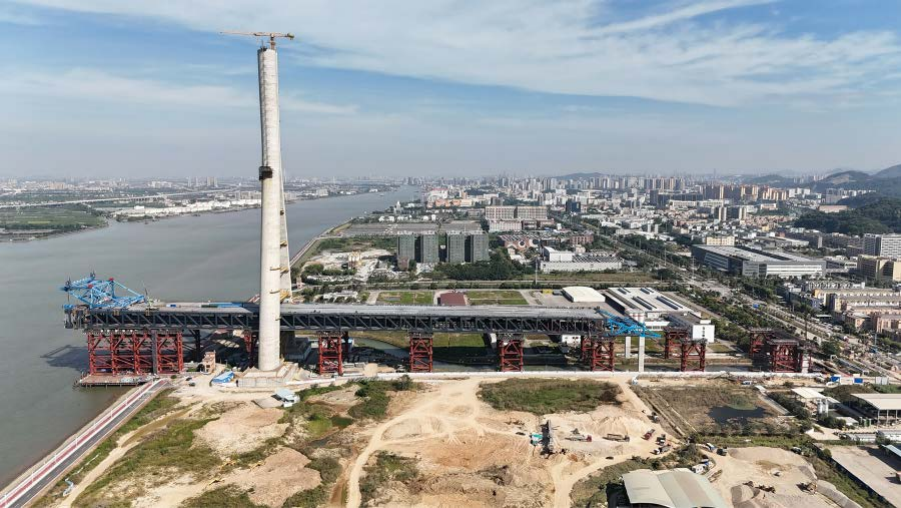
On-site incremental launching progress of Xiangshan bridge.

The Zhongshan East Ring Highway serves as the inaugural corridor linking the Shenzhen-Zhongshan Channel on the west bank of the Pearl River Estuary, playing a pivotal role within the Guangdong-Hong Kong-Macao Greater Bay Area’s highway network. The linchpin of this highway is the Xiangshan Bridge, an impressive five-span double-layer steel truss cable-stayed bridge characterized by a span configuration of (136 + 312 + 880 + 312 + 136) m. This research delves into the construction monitoring project of the Xiangshan Bridge, utilizing exclusively data derived from on-site measurements. Fig 18 provides a visual representation of the progression of the incremental launching construction activities at the Xiangshan Bridge site.

Starting from September 29, 2022, commissioned by the construction unit (Poly Changda Engineering Co., Ltd.), South China University of Technology is acting as a third-party monitoring unit to carry out construction control and scientific research on the Xiangshan Bridge, and this dissertation’s monitoring data, results, and grants are subject to the construction unit’s permission and protection.

### 5.2 Measured data collection

To achieve effective error control over the shape, it is essential to first understand the overall distribution of errors in the assembled beam segments. This understanding allows for corrective construction monitoring commands to be issued during the installation of the next beam segment. There are three methods to obtain the overall error:

(1) Comprehensive Measurement: This method involves directly measuring all beam segments on-site, the main measurement frequency arrangements and the technical parameters of the measuring instruments are given in Table 1.
(2) Recursive Prediction: The second method inputs the measured error matrix from the assembly platform into the LSTM model, which has been preliminarily trained with splines, to obtain recursive prediction results.
(3) Model Updating: The third method utilizes the comprehensive measurement errors of the entire bridge as evidence and features to update the LSTM model, addressing the issue of error accumulation in recursive predictions.

**Table 1:** Measurement arrangements and technical parameters.

	Assembly Measurement	Comprehensive Measurement
Measurement Frequency	End of each incremental launching (About once a week)	About once every 2 months
Location and Number of Measurement Points	7 points (longitudinal direction) × 6 points (transverse direction) = 42 points	All measurement points
Method	Relative elevation measurement	
Measuring Device	Leica TM50 total station Distance measuring accuracy: 0.6 mm ± 1 ppm Angle measuring accuracy: ± 0.5’‘	

**Fig 19:**
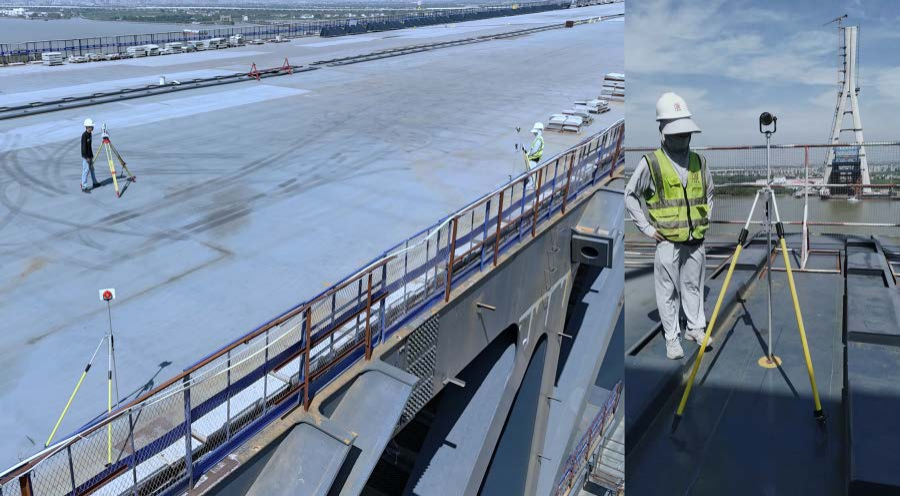
Actual measurement of shape errors.

Thus, the third method, which incorporates measured data to update the model, provides the most effective error prediction capability. Regardless of the method employed, obtaining error measurement data is essential. Fig 19 depicts the actual status of shape error measurement for the Xiangshan Bridge.

From a computational perspective, comprehensive measurement data can be used to validate the current LSTM error predictions and provide measured evidence for updating future predictions. However, obtaining accurate comprehensive measurement data demands substantial human and computational resources. In addition to the large workload involved in the measurements themselves, there are challenges associated with error separation. To mitigate measurement errors, particularly those caused by temperature fluctuations, measurements should ideally be conducted at night when temperature conditions are stable. Furthermore, the comprehensive measurement data includes deformations resulting from the deflections of the steel truss at various temporary supports. The influence of over- and under-padding on the reaction forces at the temporary piers complicates the calculation of theoretical deflection values, making the error separation process more challenging.

Table 2 shows the statistics of the shape error measurement results. Generally, the negative errors are larger than the positive errors, indicating that the actual downward deflection of the beam exceeds the estimated value. Additionally, the error values for the features are smaller than those for the labels, such as the mean and standard deviation, which reflects that the comprehensive measurement is accumulated by the process error. The manufacturing error, measured during the pre-assembly of beam segments at the plant, has a slightly larger overall error than the process error from on-site assembly. This indicates that some of the pre-assembly error was eliminated during the actual assembly process.

**Table 2:** Shape error measurement results statistics.

Statistical indicators	Process errors			Manufacturing errors	Comprehensive measurement	
	Feature1	Feature2	Feature3	Feature4	Label1	Label2
Mean	2.4	−2.0	−1.9	−0.3	−12.9	−15.9
Standard deviation	4.7	3.5	2.8	6.2	10.7	9.8
Median	0.7	0.0	−2.6	−0.1	−13.4	−17.5
Maximum	8.9	2.6	2.9	6.1	0.0	0.0
Minimum	−7.7	−8.6	−6.1	−9.4	−28.5	−28.8

### 5.3 Analysis of model results

**Fig 20:**
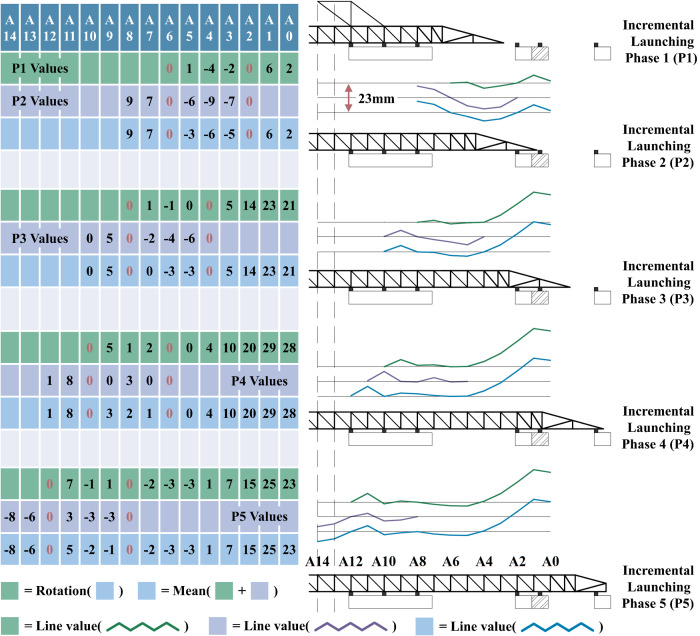
Shape error manual calculation process.

Fig 20 illustrates the manual calculation workflow for shape errors using phased monitoring results. In this process, the measured values at each phase (highlighted in purple tables) undergo zero-reset normalization at the front and rear slider positions. The zero-reset normalization refers to a geometric transformation that applies rigid-body rotation to the shape error profile, ensuring zero error at predefined reference positions (indicated by red “0” values in the tables).

The data in the green tables are derived from the zero-reset normalized row vectors of the blue tables from the preceding phase. Within each phase, the values at corresponding positions in the green and purple tables are combined via weighted averaging to generate the row vectors in the updated blue tables. The green, purple, and blue error profiles in the figure represent graphical outputs of this data processing sequence. To preserve the intrinsic geometric characteristics of the shape errors, all profiles are proportionally scaled using a fixed aspect ratio during visualization.

**Fig 21:**
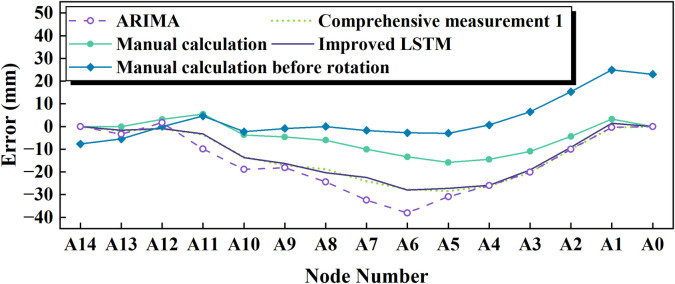
Summary Analysis of the 1st shape error in comprehensive measurement.

The cumulative post-jacking monitoring results after five incremental jacking steps were aggregated to derive the pre-rotation manual calculation outcomes in Fig 21. A marked discrepancy is evident between the manual calculations and conventional shape error measurements, whereas the modified LSTM predictions exhibit strong alignment with the latter. Due to the limited use of multiple features, the ARIMA (Autoregressive Integrated Moving Average Model, p = q = d = 1) also shows significant deviation, and once the prediction step length exceeds 1, it is largely unable to capture the error development trend. Furthermore, the LSTM-based approach demonstrates not only higher accuracy but also superior computational efficiency compared to manual methods, significantly reducing time and resource requirements. Additionally, the integration of a Kalman filter-based preprocessing module further enhances the robustness of the proposed framework by improving noise reduction and data stabilization for real-time monitoring inputs.

**Fig 22:**
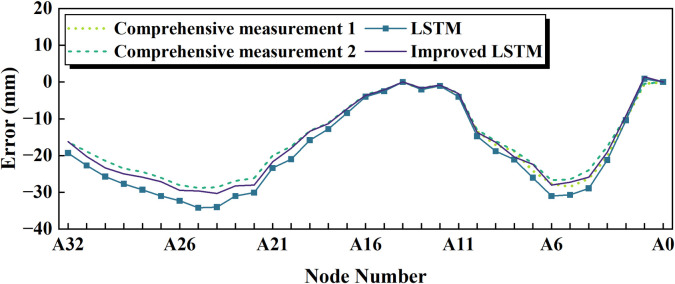
Summary analysis of the 2nd shape error in comprehensive measurement.

Fig 22 displays the 2nd shape error in comprehensive measurement. Post-Kalman-filtering processing demonstrates strong consistency with the first-phase measurements, confirming the method’s efficacy in mitigating system-induced uncertainties in monitoring outcomes. Furthermore, the modified LSTM predictions exhibit closer alignment with measure results compared to the native LSTM architecture, indicating improved capability in capturing nonlinear error patterns. These findings collectively validate the structural enhancements to the LSTM framework through the integration of a Kalman filter, a CGAN and AutoML Stacking Device, and an attention mechanism. Such hybrid modifications synergistically optimize feature extraction and temporal dependency modeling, thereby enhancing the framework’s generalization capability across dynamic monitoring scenarios.

### 5.4 Similar application cases

**Fig 23:**
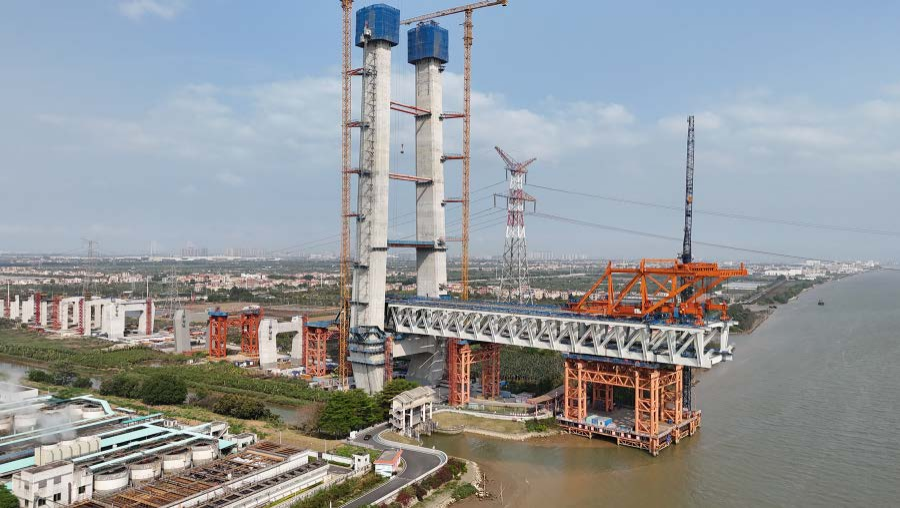
On-site incremental launching progress of Hongqili bridge.

Construction monitoring is a complex, large-scale engineering task often hindered by missing or unusual data records. The Hongqili Bridge is a project under construction by Xiamen Shenzhen Railway Guangdong Co., Ltd., with South China University of Technology entrusted by the owner to carry out construction monitoring and scientific research on the bridge. The monitoring data, results, and grants discussed in this dissertation are subject to the owner’s permission and protection. The following sections use the Hongqili Bridge, shown in Fig 23, as a case study to demonstrate the application of LSTM-related advancements.

**Fig 24:**
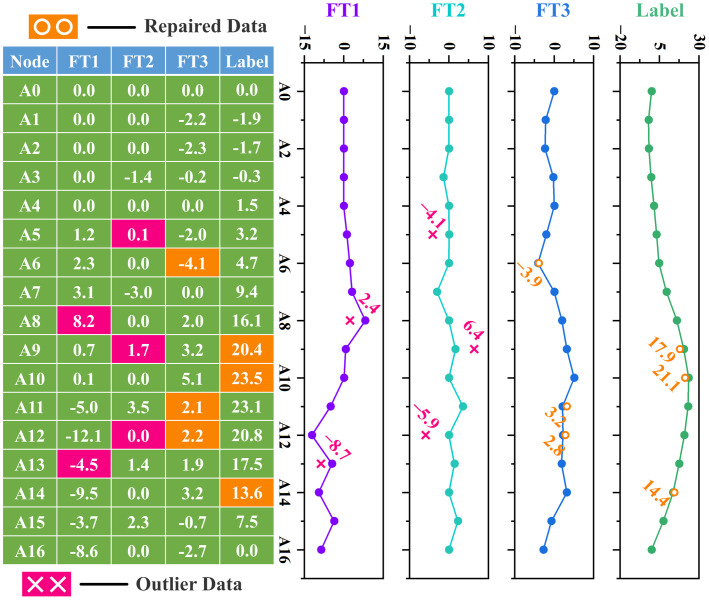
An application case of improved LSTM Data processing task.

A practical advantage of the modified LSTM architecture is its robust data imputation capability. The LSTM-based imputation works through sequence-aware prediction, utilizing intrinsic feature correlations to reconstruct missing values without the need for explicit labeling of gaps (recursive prediction). As shown in Fig 24, this method masks arbitrary columns (treated as prediction targets) while training on the unmasked columns to iteratively reconstruct missing entries. Data gaps are highlighted in different colors in the table cells, while imputed results are marked with yellow circles in the corresponding line charts. When the masked data significantly deviates from the predicted value, the program automatically flags it as an outlier, indicated by a red cross in the figure. Overall, both data repair and outlier detection have yielded strong application results.

## 6. Conclusion

This study validates the accuracy of the LSTM model in forecasting shape errors through experimental analysis of measured data, thereby providing a theoretical foundation and technical guidelines for error mitigation. This technology not only enhances the precision of controlling the construction shape of steel trusses but also has broad applications in data repair and outlier detection.

The difference between the shape error processed by manual splicing and the Comprehensive Measurement value can be as large as 15 mm, while the difference between the predicted results from the improved LSTM is only 3 mm, demonstrating that the LSTM maintains high efficiency and accuracy in processing shape error data. However, the model’s performance in long-term prediction tasks is slightly lacking, particularly when the prediction step length exceeds 2, as the prediction deviation increases significantly.

Another disadvantage is that the model is highly dependent on the amount of measured data, requiring not only a large number of cyclic and repeated sampling of basic features (such as line shapes) but also significant additional correlation features (e.g., mechanical parameters). The goal of model prediction is to reduce the workload of measurement, particularly comprehensive measurement, so maintaining a balance between prediction and measurement data is crucial. Adaptive model updating will be a key research direction to overcome these limitations, focusing on how to update the model with the least amount of measured data.

The LSTM method proposed in this paper demonstrates good generalizability and can also be applied to mid-span cantilever assembly shape error prediction and control. In this case, there are more feature types and stronger correlations, including main girder shape measurement, cable force measurement of stay cables and crane slings, member stress measurement, support reaction measurement, and temperature and humidity measurements. The fusion of these multimodal data features will enhance the accuracy and ease of shape error prediction.
